# A Crosstalk Between Brain Cholesterol Oxidation and Glucose Metabolism in Alzheimer’s Disease

**DOI:** 10.3389/fnins.2019.00556

**Published:** 2019-05-31

**Authors:** Paola Gamba, Erica Staurenghi, Gabriella Testa, Serena Giannelli, Barbara Sottero, Gabriella Leonarduzzi

**Affiliations:** Department of Clinical and Biological Sciences, University of Turin, San Luigi Hospital, Turin, Italy

**Keywords:** Alzheimer’s disease, cholesterol metabolism, oxysterols, glucose metabolism, insulin resistance, renin-angiotensin system

## Abstract

In Alzheimer’s disease (AD), both cholesterol and glucose dysmetabolism precede the onset of memory deficit and contribute to the disease’s progression. It is indeed now believed that oxidized cholesterol in the form of oxysterols and altered glucose uptake are the main triggers in AD affecting production and clearance of Aβ, and tau phosphorylation. However, only a few studies highlight the relationship between them, suggesting the importance of further extensive studies on this topic. Recently, a molecular link was demonstrated between cholesterol oxidative metabolism and glucose uptake in the brain. In particular, 27-hydroxycholesterol, a key linker between hypercholesterolemia and the increased AD risk, is considered a biomarker for reduced glucose metabolism. In fact, its excess increases the activity of the renin-angiotensin system in the brain, thus reducing insulin-mediated glucose uptake, which has a major impact on brain functioning. Despite this important evidence regarding the role of 27-hydroxycholesterol in regulating glucose uptake by neurons, the involvement of other cholesterol oxidation products that have been clearly demonstrated to be key players in AD cannot be ruled out. This review highlights the current understanding of the potential role of cholesterol and glucose dysmetabolism in AD progression, and the bidirectional crosstalk between these two phenomena.

## Introduction

Several events in the brain contribute to AD development, including neuroinflammation, oxidative stress, Aβ toxicity, NFT formation, mitochondrial dysfunction, defective insulin signaling, decreased glucose utilization, and dysregulated cholesterol homeostasis. Deficiency in insulin signaling and IR, together with alteration in glucose and cholesterol metabolism, may lead to the occurrence of neuronal dysfunction and death and, consequently, to dementia. However, the molecular mechanisms involved in AD development are not completely clear, especially as regards the interaction between the different aspects of this pathology.

Cholesterol is particularly important in the brain since it is a major component of cell membranes, thus altered cholesterol metabolism may contribute to AD development (Gamba et al., 2015). Insulin is another important regulator of brain function. It affects neuronal synaptic function and plasticity, and glucose/cholesterol metabolism in the brain (Najem et al., 2014). Substantial glucose is required during memory processing especially in the hippocampus (McNay et al., 2001) and several neurodegenerative diseases are characterized by glucose hypometabolism (Teune et al., 2010). During AD progression, glucose dysmetabolism precedes the onset of memory deficit and it is speculated to predict the disease progression (Nordberg et al., 2010).

Both brain cholesterol and glucose dysmetabolism are recognized as important features of AD, affecting the production and clearance of Aβ and tau phosphorylation, and inducing neurodegeneration (Sato and Morishita, 2015). Recently, a connection between these two processes has been highlighted; however, a more integrated understanding of the interactions between cholesterol and glucose metabolism is required in order to develop new therapeutic strategies to counteract AD. This review provides a brief summary of the rationale on the bidirectional relationship between two main risk factors in AD pathogenesis, i.e., brain cholesterol and glucose dysmetabolism due to insulin signaling deficiency.

## The Complex Role of Cholesterol in the Brain

### Brain Cholesterol Metabolism

The brain is the most cholesterol-rich organ, since it contains a quarter of the whole body non-esterified cholesterol pool (Dietschy, 2009). Cholesterol, as the main lipid component of neuronal and glial membranes and key constituent of myelin, plays essential roles in plasma membrane compartmentalization, signaling, myelination, and formation and maintenance of synapses (Petrov et al., 2017; Hussain et al., 2019).

Plasma and brain cholesterol pools are separated by two barriers: (i) the BBB, that prevents lipoprotein-bound cholesterol uptake from the circulation; (ii) the blood-CSF barrier, through which plasma is ultrafiltered to form part of the CSF. In addition, CSF interfaces the brain interstitial fluid exchanging water, ions, and other molecules (Johanson et al., 2011). Consequently, brain cholesterol metabolism is independent from that of peripheral tissues, and neurons rely on *de novo*-synthesized cholesterol delivery from astrocytes.

**Figure 1:**
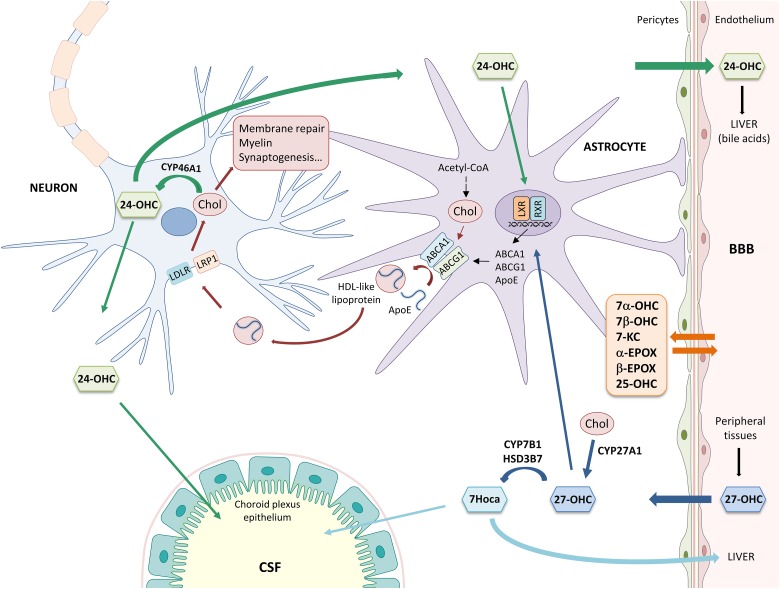
Main mechanisms involved in brain cholesterol homeostasis. α-EPOX, 5α,6α-epoxycholesterol; β-EPOX, 5β,6β-epoxycholesterol; 24-OHC, 24-hydroxycholesterol; 25-OHC, 25-hydroxycholesterol; 27-OHC, 27-hydroxycholesterol; 7α-OHC, 7α-hydroxycholesterol; 7β-OHC, 7β-hydroxycholesterol; 7Hoca, 7α-hydroxy-3-oxo-4-cholestenoic acid; 7-KC, 7-ketocholesterol; ABC, ATP-binding cassette; Acetyl-CoA, acetyl coenzyme A; ApoE, apolipoprotein E; BBB, blood-brain barrier; Chol, cholesterol; CSF, cerebrospinal fluid; HDL, high density lipoprotein; HSD3B7, 3β-hydroxy-C27-steroid dehydrogenase/isomerase; LDLR, low density lipoprotein receptor; LRP1, LDL receptor-like protein 1; LXR, liver X receptor; and RXR, retinoid X receptor.

As shown in Figure 1, cholesterol is synthesized from Acetyl-CoA through reactions catalyzed by over 20 enzymes, including HMG-CoA reductase. Newly synthesized cholesterol is loaded into lipoproteins similar to HDLs, containing the ApoE. Lipidation and secretion of ApoE are mediated by ABC transporters, such as ABCA1 and ABCG1. Then, lipoproteins are transported to neurons, where they are taken up by LDLRs and LRPs. Following receptor-mediated endocytosis, ApoE is recycled to the plasma membrane and cholesterol is used for cell membrane turnover and repair, myelin formation, synaptogenesis, and neurotransmitter release (Gamba et al., 2015; Petrov et al., 2016; Liao et al., 2017). In order to maintain brain cholesterol homeostasis, excess cholesterol is converted into oxysterols, important metabolites deriving from cholesterol enzymatic oxidation or auto-oxidation. Cholesterol is mainly converted into 24-OHC by CYP46A1, a cytochrome P-450 enzyme expressed by neurons. 24-OHC flows from the brain into the circulation across the BBB (∼99%) driven by the concentration gradient and, then, it is excreted by the liver in the form of bile acids (Björkhem et al., 2018; Dosch et al., 2019); less than 1% of 24-OHC flows into the CSF (Lütjohann et al., 1996). Brain cholesterol is also oxidized into 27-OHC by the enzyme CYP27A1, expressed by neurons and glial cells. In contrast to 24-OHC, most of the cerebral 27-OHC derives from the peripheral circulation since CYP27A1 is expressed in most of the organs and tissues (Marwarha and Ghribi, 2015). 27-OHC is indeed one of the major oxysterols in human circulation and its flux into the brain is likely driven by the concentration gradient, maintained by the high rate of its brain metabolism into 7Hoca by CYP7B1 and HSD3B7; subsequently, 7Hoca is eliminated in the systemic circulation and in the CSF (Meaney et al., 2007; Saeed et al., 2014; Björkhem et al., 2018). Both 24-OHC and 27-OHC can, in turn, regulate cholesterol synthesis and transport from glia to neurons by acting on the nuclear LXR, that regulates the expression and synthesis of ApoE and ABCA1/ABCG1 (Czuba et al., 2017). In addition to 24-OHC and 27-OHC, other oxysterols are present in the brain (Testa et al., 2016). Besides enzymatic oxidation, cholesterol auto-oxidation can be induced by different compounds, such as lipid peroxides, free radical species, and metal cations, resulting in the formation of various oxysterols. Among them, 7α-OHC, 7β-OHC, 7-KC, 25-OHC, α-EPOX, and β-EPOX are the most representative. Both 7α-OHC and 25-OHC can also derive from cholesterol enzymatic oxidation, respectively by CYP7A1 and CH25H (Leoni and Caccia, 2013). These oxysterols flow from the brain into the systemic circulation and *vice versa*, crossing the BBB (Figure 1).

### The Involvement of Oxysterols in Alzheimer’s Disease

There has been growing evidence about the involvement of altered cholesterol metabolism in AD (Wood et al., 2014; Zarrouk et al., 2014, 2018; Gamba et al., 2015; Testa et al., 2018a). The AD brain, in particular the cortex and the hippocampus, is characterized by synaptic dysfunction, extracellular deposits of Aβ as senile plaques, and intracellular inclusions consisting of hyperphosphorylated tau protein as NFTs, all factors contributing to neuronal loss (Querfurth and LaFerla, 2010).

The presence of oxysterols in the brain could be one of the factors contributing to AD progression. It has been shown that some oxysterols (e.g., 27-OHC, 7β-OHC, and 7-KC) significantly increase in AD brains compared to healthy brains; in contrast, 24-OHC brain levels decrease likely due to neuronal loss (Hascalovici et al., 2009; Testa et al., 2016).

Concerning 27-OHC, its increased flux into the brain can be favored by hypercholesterolemia that induces oxidative stress, thus altering BBB permeability (Heverin et al., 2004; Dias et al., 2014). Moreover, under oxidative stress and inflammatory conditions, brain cholesterol metabolism into 27-OHC increases because the enzyme CYP27A1 is highly expressed by glial cells. Both these mechanisms cause the increase of 27-OHC/24-OHC brain ratio (Marwarha and Ghribi, 2015). 27-OHC has been observed to promote pro-inflammatory molecule release (Testa et al., 2014), to increase Aβ levels (Prasanthi et al., 2009; Gamba et al., 2014), in human neuroblastoma cell lines and both Aβ and hyperphosphorylated tau levels in rabbit organotypic hippocampal slices (Marwarha et al., 2010). Moreover, 27-OHC has been recently demonstrated to impact on lysosomal membrane permeabilization and pyroptosis in co-cultured SH-SY5Y and C6 cells (Chen et al., 2019). In addition, increased Aβ plaques were found in the hippocampus of 27-OHC-treated mice (Zhang et al., 2018), and 27-OHC has been shown to induce synaptic dysfunction and to impair neuron morphology (Merino-Serrais et al., 2019).

As regards 24-OHC, contrasting effects have been reported: on the one hand it promotes neuroinflammation, Aβ peptide production, oxidative stress, and cell death in neuronal cell lines (Gamba et al., 2011, 2014; Yamanaka et al., 2011; Testa et al., 2014); on the other hand, it has been reported to play an important role in regulating brain cholesterol metabolism via LXR, and to exert beneficial effects such as preventing tau hyperphosphorylation in SK-N-BE cells, suppressing Aβ production in SH-SY5Y cells, and regulating synaptic function in rat hippocampal neurons and slices (Paul et al., 2013; Urano et al., 2013; Testa et al., 2018b). These opposite effects may depend on 24-OHC concentration, since low concentrations (1–10 μM) seem to induce adaptive responses and beneficial effects in neuronal cell lines as discussed by Testa et al. (2018a).

## The Interplay Between Cholesterol and Glucose Metabolism in the Brain

### The Role of Oxysterols in Brain Insulin Resistance

Insulin is an important regulator of brain cell function and metabolism: it affects neuronal synaptic function and plasticity and regulates both glucose and cholesterol metabolism. Like in peripheral tissues, insulin signaling in the brain is mediated by the binding of insulin to its receptor. Consequently, insulin receptor auto-phosphorylation leads to the phosphorylation of the IRS family, of which IRS1 is the best characterized. IRS1 activates two important signaling pathways: the PI3K/Akt pathway and the MAPK cascade (Akter et al., 2011). The activation of the insulin signaling cascade leads to the translocation of the insulin-sensitive GLUT4 to the plasma membrane to favor glucose uptake during memory-related cognitive functions (McEwen and Reagan, 2004).

However, the insulin-mediated glucose uptake in the brain is not as significant as in the periphery. Indeed, brain glucose uptake is also regulated by the cerebral RAS, which is essential for several brain functions, such as learning, memory, emotional responses, and processing of sensory information. A significant reduction of RAS activity has been reported in the AD brains (Mateos et al., 2008, 2011a,b). The downstream peptide Ang IV binds to its receptor, known as IRAP, which is localized in specialized vesicles containing GLUT4 within hippocampal neurons, as well as throughout other brain regions. This binding inhibits IRAP activity, thus preventing the cleavage of memory-enhancing peptides, and activates GLUT4 favoring glucose uptake, thus preserving cognitive functions (Wright and Harding, 2008).

**Figure 2:**
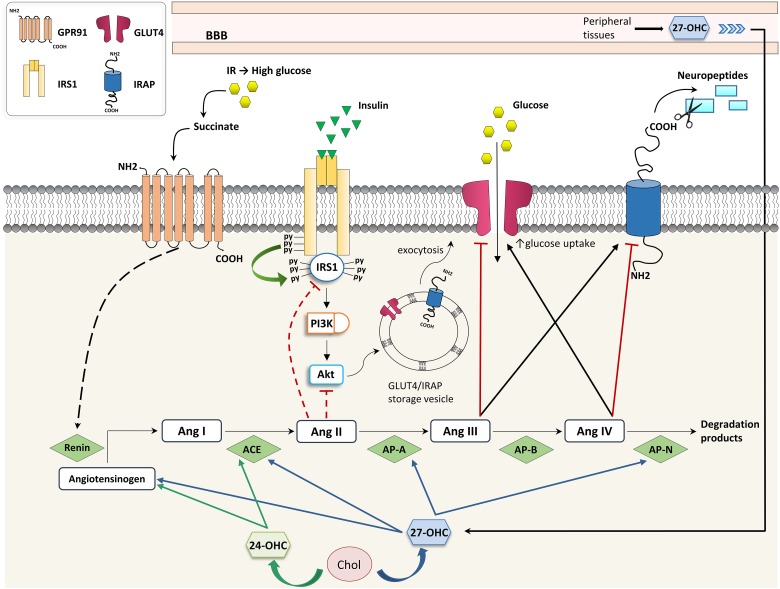
Effects of oxysterols on RAS- and insulin-dependent glucose uptake. 24-OHC, 24-hydroxycholesterol; 27-OHC, 27-hydroxycholesterol; ACE, angiotensin I-converting enzyme; Ang, angiotensin; AP, aminopeptidase; BBB, blood-brain barrier; Chol, cholesterol; GLUT, glucose transporter; GPR91, G-protein-coupled receptor 91; IR, insulin resistance; IRAP, insulin-regulated aminopeptidase; IRS, insulin receptor substrate; and PI3K, phosphoinositide 3-kinase.

Recently, a molecular link was demonstrated among cholesterol metabolism, brain glucose uptake, and the brain RAS, all of which are affected in neurodegenerative diseases. Besides being a link between hypercholesterolemia and the increased AD risk, 27-OHC is considered a biomarker for the reduced brain glucose metabolism in AD since it is able to increase brain RAS activity, thus impairing neuronal glucose uptake (Figure 2). In particular, 27-OHC is involved in the reduction of glucose uptake in the brain by modulating the activity of IRAP and GLUT4. To do this, 27-OHC increases the expression of two main factors involved in the cerebral RAS: AP-A, which transforms Ang II into Ang III, and AP-N, which degrades Ang IV (Ismail et al., 2017). Since Ang III and Ang IV have opposite effects (Ang III inhibits GLUT4 and activates IRAP and, *vice versa*, Ang IV activates GLUT4 and inhibits IRAP), it can be assumed that 27-OHC excess in the brain, as in the case of AD, may reduce brain glucose uptake which has a major impact on brain functioning (Figure 2). In this connection, *in vivo* experiments demonstrated that intracerebroventricular injection of 10 μM 27-OHC in WT mice significantly reduces the levels of GLUT4 and increases the levels of AP-A, AP-N and IRAP in the hippocampus. Moreover, a decrease in GLUT4 levels and an enhancement in IRAP levels were observed in cortical and hippocampal primary neurons treated with 1 μM 27-OHC (Ismail et al., 2017). The activation of IRAP by 27-OHC causes the cleavage of neuropeptides and thus contributes to memory deterioration (Lew et al., 2003). These results are supported by the fact that *CYP27A1* overexpressing mice show decreased glucose metabolism and memory deficit (Ismail et al., 2017). *In vitro* experiments also demonstrated that treatments of rat primary neurons, astrocytes, and human neuroblastoma cells with 1–10 μM 27-OHC stimulate the production of angiotensinogen, the precursor of Ang I. Moreover, in AD the activity of ACE correlates with 27-OHC levels both in plasma and CSF (Mateos et al., 2011a), although ACE levels have been shown to be reduced in the CSF (Miners et al., 2009).

Several effects exerted by 27-OHC on brain RAS have been observed to be mediated by LXRβ, since 27-OHC is a good LXR ligand (Ismail et al., 2017). However, besides 27-OHC, other oxysterols have been identified as endogenous ligands for LXR, including 24S-OHC (Nagy et al., 2012). In fact, both 24S-OHC and 27-OHC regulate the brain RAS in primary neurons and astrocytes through a LXR-dependent mechanism, by upregulating angiotensinogen, ACE and Ang II type 1 receptors, all involved in neuronal plasticity, learning, and memory (Mateos et al., 2011b). In addition to LXRβ, also LXRα regulates glucose uptake since the LXRα binding site has been found in the *GLUT4* promoter (Dalen et al., 2003).

In addition, the expression of *GLUT4*, together with the expression of other genes involved in glucose metabolism control, may be regulated by PPARγ (Komers and Vrána, 1998). This nuclear receptor is also involved in the increase of LRP1, a member of the LDL receptor family involved in cholesterol metabolism but also in AD pathogenesis (Shinohara et al., 2017). LRP1 participates in Aβ uptake and metabolism, and in amyloid precursor protein trafficking (Xue-Shan et al., 2016). Moreover, LRP1 is strongly associated to IR because it is involved in the insulin receptor trafficking and intracellular signaling, as well as in glucose uptake in several tissues, but mainly in the brain (Actis Dato and Chiabrando, 2018). In this regard, neuronal LRP1 deficiency leads to a reduced insulin receptor localization in the plasma membrane, an impaired insulin signaling, and decreased glucose uptake due to the lack of GLUT3 and GLUT4 (Liu C.C. et al., 2015).

Both the nuclear receptors PPARγ and LXRs are also implicated in the regulation of lipid metabolism. In this context, *CYP27A1* gene expression, regulated by PPARγ and LXR signaling, results in increased levels of 27-OHC, which in turn up-regulates PPARγ and LXR-dependent processes (Szanto et al., 2004; An et al., 2017). Moreover, an oxysterol mixture compatible with that detectable in human hypercholesterolemic plasma, but not unoxidized cholesterol, has been shown to upregulate PPARγ (Leonarduzzi et al., 2010). In addition, oxidized derivatives of fatty acids, such as 9- and 13-hydroxyoctadecadienoic acid, both oxidized LDL components, activate PPARγ in macrophages (Nagy et al., 2012). Furthermore, macrophage-specific *PPARγ* knockout mice easily develop diet-induced obesity, glucose intolerance and IR (Hevener et al., 2007; Odegaard et al., 2007).

Brain IR is defined as the inadequate response to insulin by target cells and it has been considered a key feature in AD development since it is highly related to tau pathology. IR is, indeed, associated with higher tau levels in the CSF (Starks et al., 2015), and CSF tau predicts changes in brain glucose metabolism (Dowling et al., 2015). It has been observed that in the AD brain there are lower levels of insulin and of insulin receptors, resulting in reduced PI3K/Akt signaling (Schubert et al., 2003, 2004) and GSK3β activation, responsible for NFT formation (Doble and Woodgett, 2003).

A direct crosstalk between high glucose levels induced by IR and RAS has been highlighted in kidneys. In particular, hyperglycemia induced by IR modulates RAS by leading to renin release through the binding of succinate to its receptor GPR91 (Peti-Peterdi et al., 2008); *vice versa*, RAS contributes to IR because Ang II impairs insulin signaling through IRS1 or PI3K/Akt inhibition, as shown in Figure 2 (Andreozzi et al., 2004). At present there is no evidence that this regulatory network exists also in the brain, but it has been demonstrated that 27-OHC and 24-OHC interfere in the brain’s insulin-dependent glucose uptake through RAS.

The role of the HO-1/BVR-A system in the occurrence of IR in the brain, in particular in AD, is gaining attention (Barone and Butterfield, 2015).

The enzyme HO-1 is markedly overexpressed in cortical and hippocampal neurons and astroglia, and colocalizes with senile plaques and NFTs (Schipper et al., 1995). The upregulation of HO-1, in particular by the astrocytic compartment, may confer cytoprotection by enhancing the break-down of prooxidant heme to the radical scavenging biliverdin and bilirubin. However, under certain conditions, heme-derived iron and CO may exacerbate intracellular oxidative stress by provoking free radical generation within mitochondria and other subcellular organelles. The interplay between brain HO-1 and cholesterol homeostasis may have important implications in the pathogenesis of AD. In this connection, it has been demonstrated that HO-1 levels increase in the AD brain in parallel with the increased levels of oxysterols; indeed, HO-1 overexpression suppresses total cholesterol levels by favoring LXR-mediated cholesterol efflux, and enhances oxysterol formation (Vaya and Schipper, 2007; Hascalovici et al., 2014).

Brain IR may be due to increased phosphorylation of IRS1 on specific residues. In this connection, BVR-A is the kinase that phosphorylates and inhibits IRS1, consequently inhibiting the insulin signaling. For this reason, BVR-A is considered a novel mediator of IR. Interestingly, oxidative stress affects BVR-A function resulting in the impairment of the insulin signaling in AD subjects (Barone et al., 2011, 2016).

### The Impact of High Fat Diet-Induced Hypercholesterolemia on Brain Insulin Resistance

As one of the most cholesterol-rich organs, brain cholesterol homeostasis is tightly regulated; however, there is growing evidence that the brain lipid profile may be modified by HFD-induced hypercholesterolemia (Czuba et al., 2017).

In this connection, in AD and aging animal models it has been observed that HFD induces cognitive decline (Pancani et al., 2013; Knight et al., 2014). Long-term exposure to HFD results in the increase of plasma cholesterol and, most importantly, disturbs brain cholesterol homeostasis leading to Aβ accumulation, hyperphosphorylation of tau, and neuronal death (Vance, 2006). Moreover, the HFD triggers astrocytic activation in the murine hippocampi and increases the expression of proteins involved in cholesterol transport across brain cell membranes, such as ApoE, thus HFD has a great impact on brain cholesterol homeostasis (Chen et al., 2016). Reactive astrocytes release various inflammatory mediators, that can promote senile plaque and NFT formation that, in turn, contribute to the redox imbalance and inflammation. Cholesterol fed rabbits exhibit high levels of both reactive oxygen species and antioxidant enzyme HO-1 in the brain, and the increment of HO-1 correlates well with oxysterol levels (Hascalovici et al., 2014). It has also been shown that the brain levels of 27-OHC, transported from the systemic circulation, increased in high cholesterol fed rabbits, thus leading to neurodegeneration in the hippocampus (Brooks et al., 2017).

Besides the increased risk of AD induced by HFD because of brain cholesterol dysmetabolism (Stapleton et al., 2008), it has also been demonstrated that HFD induces hepatic IR and impairment of synaptic plasticity (Liu Z. et al., 2015). Additionally, *in vivo* studies demonstrated that HFD-induced peripheral IR and *apoE*ε*4* gene variant synergistically impair cerebral insulin signaling (Zhao et al., 2017). The influence of HFD on the development of brain IR has been demonstrated by the presence, in the hippocampi of HFD fed mice, of elevated levels of phospho-IRS1 (Ser616) (Arnold et al., 2014), phospho-Akt (Ser473), and phospho-GSK3β (Ser9) (Spinelli et al., 2017). Both short-term diet, with very high fat content, and long-term diet, with moderate fat, interfere with the insulin signaling pathways and induce IR in the brain (Arnold et al., 2014).

Furthermore, few studies highlight the importance of serum cholesterol in brain glucose uptake. Higher midlife serum total cholesterol levels are associated, in humans, with lower metabolic glucose rate in brain areas affected by AD, such as precuneus, parietotemporal, and prefrontal regions, but also in frontal regions that are commonly affected by normal aging (Reiman et al., 2010). Moreover, high levels of blood cholesterol enhance RAS activity in the brain: high cholesterol fed mice show increased levels of the precursor angiotensinogen and of ACE (Mateos et al., 2011b). Moreover, HFD fed mice exhibit increased IRAP catalytic activity in the brain (Ismail et al., 2017).

### Insulin Resistance Regulates Cholesterol Metabolism in the Brain

The crosstalk between cholesterol dysmetabolism and IR is bidirectional: not only hypercholesterolemia and altered cholesterol homeostasis affect IR, but also IR may, conversely, affect cholesterol metabolism; in fact, insulin can activate the transcription factors SREBPs involved in cholesterol biosynthesis (Suzuki et al., 2010). In addition, insulin increases cholesterol biosynthesis in SH-SY5Y and N2a cells, by upregulating 24-dehydrocholesterol reductase, and HMG-CoA reductase through SREBP2, whereas Aβ-induced IR leads to dysregulation of cholesterol homeostasis (Najem et al., 2016). Moreover, insulin-deficient diabetes leads to a reduced cholesterol synthesis in the brain due to lower expression of SREBP2 and of its downstream genes in the hypothalamus and in other brain regions, resulting in altered synaptic formation, and function (Suzuki et al., 2010, 2013). Conversely, cholesterol depletion in GT1-7 hypothalamic neuron-derived cells contributes to IR, alters autophagy, and enhances apoptosis induced by cytotoxic stress (Fukui et al., 2015).

## Conclusion

Disruption of cholesterol and glucose metabolism are key players in AD onset and progression, however, the crosstalk between these two phenomena is not yet clear. Despite the important evidence regarding the role of certain oxysterols in regulating glucose uptake by neurons, it would be crucial to deepen their role in modulating the insulin signaling pathway in the brain in order to develop new strategies aimed at preventing or delaying AD development.

## Author Contributions

All authors listed have made a substantial, direct and intellectual contribution to the work, and approved it for publication.

## Conflict of Interest Statement

The authors declare that the research was conducted in the absence of any commercial or financial relationships that could be construed as a potential conflict of interest.

## Abbreviations

α-EPOX: 5α,6α-epoxycholesterol
β-EPOX: 5β,6β-epoxycholesterol
24-OHC: 24-hydroxycholesterol
25-OHC: 25-hydroxycholesterol
27-OHC: 27-hydroxycholesterol
7α-OHC: 7α-hydroxycholesterol
7β-OHC: 7β-hydroxycholesterol
7Hoca: 7α-hydroxy-3-oxo-4-cholestenoic acid
7-KC: 7-ketocholesterol
Aβ: amyloid β
ABC: ATP-binding cassette
ACE: angiotensin I-converting enzyme
Acetyl-CoA: acetyl coenzyme A
AD: Alzheimer’s disease
Ang: angiotensin
AP: aminopeptidase
ApoE: apolipoprotein E
BBB: blood-brain barrier
BVR-A: biliverdin reductase A
CH25H: cholesterol 25-hydroxylase
CSF: cerebrospinal fluid
CYP27A1: cholesterol 27-hydroxylase
CYP46A1: cholesterol 24-hydroxylase
CYP7A1: cholesterol 7α-hydroxylase
CYP7B1: oxysterol 7α-hydroxylase
GLUT: glucose transporter
GPR91: G-protein-coupled receptor 91
GSK3β: glycogen synthase kinase 3β
HDL: high density lipoprotein
HFD: high fat diet
HMG-CoA: 3-hydroxy-3-methylglutaryl-coenzyme A
HO-1: heme oxygenase 1
HSD3B7: 3β-hydroxy-C27-steroid dehydrogenase/isomerase
IR: insulin resistance
IRAP: insulin-regulated aminopeptidase
IRS: insulin receptor substrate
LDLR: low density lipoprotein receptor
LRP: LDL receptor-like protein
LXR: liver X receptor
MAPK: mitogen-activated protein kinase
NFT: neurofibrillary tangle
PI3K: phosphoinositide 3-kinase
PPARγ: peroxisome proliferator-activated receptor γ
RAS: renin-angiotensin system
SREBP: sterol-regulatory element-binding protein.
